# Bureau of Animal Industry

**Published:** 1885-01

**Authors:** 


BUREAU OF ANIMAL INDUSTRY.—The new bureau in the Agricultural Department is doing good work. Its field of operation is a wide and important one—the sanitary condition of 42,500,000 cattle valued at $1,105,715,703.

The enormous extent of this industry, and the terrible results that might follow the introduction and spread of a contagious disease among such an immense herd, were forcible reminders of the necessity of strict vigilance on the part of those who were placed in charge.

Commissioner Loring co-operated with Prof. Salmon in this work when an epidemic threatened, and the cattle dealers throughout the country gave their aid, giving information which was of great value to the industry.

Experiments were made at Washington to test the contagiousness of pleuro-pneumonia, the results proving that only cattle in poor condition are attacked when exposed to the infection.

Later, the bureau ordered the inspection of the stables, stock yards, and slaughter-houses in this city. In 756 stables, 26 out of 3,318 cattle were affected with pleuro-pneumonia. On Long Island 325 diseased cattle were found out of 10,072 in 1413 stables. In Staten Island 12 out of 3,857 in 555 stables. In Jersey City 8 out of 180 in 13 stables. The New York offal dock developed 20 cases out of 63 inspected; the slaughter-houses 14 out of 76 inspected, whilst Brooklyn showed 100 in 868 cattle in 60 stables.

In extirpating these diseases isolation proved a marked success. The report of the bureau to Congress will be of special interest.

